# An expanding HIV epidemic among older adults in Ukraine: Implications for patient-centered care

**DOI:** 10.1371/journal.pone.0256627

**Published:** 2021-09-30

**Authors:** Julia Rozanova, Oleksandr Zeziulin, Katherine M. Rich, Frederick L. Altice, Tetiana Kiriazova, Irina Zaviryukha, Tetiana Sosidko, Komal Gulati, Constance Carroll, Sheela V. Shenoi

**Affiliations:** 1 Yale University School of Medicine, Section of Infectious Diseases, New Haven, CT, United States of America; 2 Yale University School of Public Health: Center for Interdisciplinary Research on AIDS (CIRA), New Haven, CT, United States of America; 3 Ukrainian Institute on Public Health Policy, Kyiv, Ukraine; 4 Harvard Medical School, Boston, MA, United States of America; 5 Centre of Excellence of Research in AIDS (CERiA), University of Malaya, Kuala Lumpur, Malaysia; 6 Yale University School of Public Health, Division of Epidemiology of Microbial Diseases, New Haven, CT, United States of America; 7 100%-Life: All-Ukrainian Network for People Living with HIV, Kyiv, Ukraine; 8 Lewis Katz School of Medicine, Temple University, Philadelphia, PA, United States of America; Medical University of Warsaw, POLAND

## Abstract

**Introduction:**

The Eastern Europe and Central Asian (EECA) region has the highest increase in HIV incidence and mortality globally, with suboptimal HIV treatment and prevention. All EECA countries (except Russia) are low and middle-income (LMIC). While LMIC are home to 80% of all older people living with HIV (OPWH), defined as ≥50 years, extant literature observed that newly diagnosed OPWH represent the lowest proportion in EECA relative to all other global regions. We examined HIV diagnoses in OPWH in Ukraine, a country emblematic of the EECA region.

**Methods:**

We analysed incident HIV diagnoses from 2015–2018 and mortality trends from 2016–2018 for three age groups: 1) 15–24 years; 2) 25–49 years; and 3) ≥50 years. AIDS was defined as CD4<200cells/mL. Mortality was defined as deaths per 1000 patients newly diagnosed with HIV within the same calendar year. Mortality rates were calculated for 2016, 2017, and 2018, compared to age-matched general population rates, and all-cause standardized mortality ratios (SMRs) were calculated.

**Results:**

From 2015–2018, the proportion of OPWH annually diagnosed with HIV increased from 11.2% to 14.9% (p<0.01). At the time of diagnosis, OPWH were also significantly (p<0.01) more likely to have AIDS (43.8%) than those aged 25–49 years (29.5%) and 15–24 years (13.3%). Newly diagnosed OPWH had the same-year mortality ranging from 3 to 8 times higher than age-matched groups in the Ukrainian general population.

**Conclusions:**

These findings suggest a reassessment of HIV testing, prevention and treatment strategies in Ukraine is needed to bring OPWH into focus. OPWH are more likely to present with late-stage HIV and have higher mortality rates. Re-designing testing practices is especially crucial since OPWH are absent from targeted testing programs and are increasingly diagnosed as they present with AIDS-defining symptoms. New strategies for linkage and treatment programs should reflect the distinct needs of this target population.

## Introduction

Due to advances in antiretroviral therapy (ART), HIV has emerged as a life-long, chronic disease with a growing focus on comorbidities that increase with age [1]. The WHO 2016 strategy for person-centred care for people living with HIV (PWH) acknowledge that treatment goals are not limited to viral suppression but also include addressing healthy aging [2, 3]. Alongside the 90-90-90 targets, high-income countries (HIC) are embracing the need to address the 4^th^ 90: healthy or successful aging with HIV [4], whereby PWH may attain both the life expectancy of peers without HIV and optimal health-related quality of life [5–8]. High-income settings like the U.S. define those ≥50 years with HIV as older people living with HIV (OPWH) [9], with 80% of such individuals residing in low/middle income countries (LMIC) [10].

Unfortunately, achieving the three 90s for OPWH lags in LMIC and discussion of the 4^th^ 90 is conspicuous in its absence. While achieving quality of life for PWH is not on the agenda in most LMICs, much of this omission about OPWH is the dearth of information from this population in LMIC, thus thwarting HIV prevention and treatment strategies in this group [11–13]. HIV research and policy agendas in LMIC have prioritised risk factors including substance use [14] and sexual behaviours among younger adults [15], but have thus far overlooked OPWH [16]. Moreover, while HIV care in LMIC targets younger age groups, clinicians receive relatively little training about the distinct needs and risks among older people, including managing comorbidities and risky sexual behaviours, leading to lower screening and case detection rates in this group [11].

Eastern Europe and Central Asia (EECA) remains the region with the highest increases in HIV incidence and mortality, surpassing Southern and Eastern Africa [17]. All the countries in EECA are LMIC (except Russia). Ukraine’s HIV epidemic, emblematic of the EECA region, is the worst in Europe with a prevalence of 1.2% and the second-largest in EECA with 240,000 PWH [18, 19]. In 2017, Ukraine committed to 90-90-90 UNAIDS HIV targets [18]. While Ukraine has made some progress towards meeting these targets, ART coverage was only 52% overall in 2019 [20]. Furthermore, efforts to reach these targets in HIV treatment and prevention in Ukraine have focused on younger people, especially those 15–24 years [21–25]. Despite an aging population where adults ≥50 years constitute 37% of all Ukrainians [26–29], this group is neglected by HIV prevention and treatment initiatives [30]. While extant literature marked an increase in the number of OPWH in Southern Africa, North America, and high-income European countries [31, 32], the literature from EECA suggested that the proportion throughout EECA was unchanged and the lowest relative to other regions [33]. Observations by clinicians and epidemiologists in Ukraine were not aligned with this report and we therefore sought to examine the trends in HIV incidence and mortality by age in Ukraine using multiple available databases.

## Materials and methods

### Setting

Ukraine is a LMIC with increasing HIV incidence [34]. HIV care, including ART, is provided free at governmental HIV clinics throughout the country. In December 2016, CD4 count criteria of <500cells/mL for ART was eliminated and access was expanded to include all PWH [35, 36].

### Data sources

To characterize the HIV epidemic in Ukraine among OPWH we gathered and assessed all available largescale HIV data sources. The key source was Ukraine’s largest electronic clinical surveillance system CasePlusPlus (CPP), maintained by 100%Life, the largest non-profit organization providing services to PWH, and included information on ~155,000 individuals diagnosed with HIV in Ukraine [37] and registered for HIV care at a clinic [38, 39]. By 2018, 100%Life provided services across 270 HIV clinical centers nationwide. While all the medical staff salaries in these clinics were paid by the government, their ancillary HIV care team members like social workers and case managers were employed by 100%Life. The NGO also had as part of its requirement to international funders, to ensure accurate patient data collection and entry into the CPP database. Starting in January 2016, the CPP database added information on CD4 count at the time of diagnosis, and whether PWH died in the same calendar year they were diagnosed (based on social workers’ verification of clients’ deaths). The CPP database was used to assemble the aggregate data tables used in this analysis for the years 2015, 2016, 2017, and 2018; including the number of PWH newly diagnosed with HIV by 5-year age groups, the number of PWH who had CD4≤200 cells/mL at the time of diagnosis, and the number of newly diagnosed PWH who died.

The Ukrainian Center for Public Health (UCPH), the surveillance arm of the Ukrainian Ministry of Health, maintains another database. It maintains the most comprehensive HIV information nationwide, collecting data from all organizations that diagnose and treat HIV in Ukraine. This database is mandated by legislation and all sites must comply with reporting. While the UCPH database documents all HIV diagnoses in the country, it only began collecting CD4 count at HIV diagnosis and mortality data in July 2018. Thus, we use the UCPH national database to 1) assess the representativeness of CPP data (which has more granular data on CD4 and mortality) and 2) provide national descriptive statistics on the HIV epidemic. The UCPH provided aggregate data on the annual number of new HIV diagnoses for three age groups, stratified by sex: 15–24 years; 25–49 years; and ≥50 years (i.e., older adults) for the years 2015 (N = 12,893), 2016 (N = 14,249), 2017 (N = 15,580), and 2018 (N = 15,671) [25, 32]. UCPH also provided aggregate data on the HIV transmission categories from 2015–2018. The CPP database increasingly (41% in 2015 to 71% in 2018) incorporated more new HIV cases reported from the UCPH HIV surveillance system over time [39, 40]. Importantly, patient-level data was not available from either database. For standardized mortality analysis we used publicly available data from the Ukraine general population for 2016, including crude death rates published by the Ukrainian Bureau of Statistics [41].

### Timeframe of collecting and assembling the data

Aggregate data tables used in the analyses were assembled from Sept 1, 2019 –Oct 31, 2019. The data aggregated in the tables had been collected by the respective organizations (100% Life and the Ukrainian Center for Public Health) from Jan 1, 2015-Dec 31, 2018.

### Analysis

Using CPP data, we examined trends in new HIV diagnoses for the three age groups: 15–24 years, 25–49 years, and ≥50 years [25, 32]. We performed Somer’s D/RC and Cochran-Armitage tests to analyse trends in new HIV diagnoses among these age groups. Bi-variate statistical analyses were performed using Chi-square tests to compare proportions having AIDS (defined as CD4<200 cells/mL) at time of diagnosis by age group and by sex, with significance set as p<0.05. This definition of AIDS is consistent with the Centers for Disease Control (CDC) and National Institutes of Health (NIH) recommendations that CD4<200 cells/mL is the key immunological marker for AIDS in surveillance data [42]. While not available in surveillance data, the Ukrainian clinical protocol for HIV diagnosis and treatment uses WHO-proposed staging system, where AIDS is 4^th^ clinical stage when opportunistic infections are diagnosed [43]. All-cause SMRs were calculated by 5-year age groups of newly diagnosed PWH for 2016, 2017, and 2018, and compared to age-matched Ukraine general population crude mortality rates (CMR). To evaluate the representativeness of CPP data to UCPH national surveillance data, we assessed gender and age differences using chi-square goodness-of-fit testing. SAS 9.4 was used for all data analysis.

### Ethics review approval

No personal identifiers were used in this analysis and the study was approved by institutional review boards at [anonymized] University and the Ukrainian Institute [anonymized].

## Results

We examined whether older adults comprise a growing share of new annual HIV diagnoses. From 2015–2018 the proportion of older adults (≥50 years of age) among new HIV cases increased significantly each year (11.2% in 2015, 12.4% in 2016, 13.27% in 2017 and 14.9% in 2018; p<0.01 for trend). Moreover, CPP data showed that in the three-year period from 2016–2018, Ukrainian adults ≥50 years were significantly more likely to have AIDS (CD4<200) at diagnosis (43.8%) than 25–49 years (29.4%) and 15–24 years (13.2%) (p<0.001); this relationship remained similar for each year (Table 1). There were no significant sex differences observed in any age group.

**Table 1:** Percent of sample with CD4 count <200 cells/mL at time of HIV diagnosis by age.

Year	Age Group			p-value
	15–24	25–49	50+	
2016	15.0%	34.9%	48.2%	<0.001***
2017	12.4%	27.1%	40.8%	<0.001***
2018	12.9%	26.8%	37.6%	<0.001***

Individuals diagnosed with HIV were found to have significantly higher mortality rates within 1 year of diagnosis than the general population, with the SMRs in 2016, 2017, and 2018 all being over 6.0 (p<0.001; Table 2). SMR analyses (Tables 2 and 3) indicated that from 2016 to 2018, mortality among newly diagnosed PWH ≥50 years was between 3.8 and 8.1 times higher relative to the age-matched Ukrainian general population (p<0.0001). Mortality was significantly higher among OPWH diagnosed with HIV from 2016–2018 compared to both younger groups diagnosed with HIV and to age-matched peers in the general population. In 2018, 6.8% of OPWH died the same year that they were diagnosed with HIV, and they were significantly more likely to die the year they were diagnosed. OPWH were also two-fold (2.2) and nine-fold (9.3) more likely to die within the calendar year than those 25–49 years (p<0.001) and 15–24 years (p<0.001), respectively.

**Table 2:** Standardized mortality ratios by age group, 2016–2018.

Age Group	Deaths among recently diagnosed	Total HIV diagnoses	Ukraine General Population Crude Mortality	Standardized Mortality Ratio	Lower 95% CI	Upper 95% CI	p-value
**Year: 2018**							
20 - <25	7	462	84.6	17.9	4.6	31.2	0.0125
25 - <50	437	8119	366.6	14.8	13.4	16.2	<0.0001***
≥50	154	1465	2679.9	8.1	6.8	9.4	<0.0001***
**Total**	**598**	**10064**	**1392.5**	**12.3**	**11.3**	**13.2**	<0.0001***
**Year: 2017**							
20 - <25	3	519	84.6	6.8	0.0	14.6	0.1393
25 - <50	201	8767	366.6	6.6	5.7	7.6	<0.001***
≥50	75	1277	2184.1	4.7	3.6	5.7	<0.001***
**Total**	**279**	**10569**	**1167.1**	**6.0**	**5.3**	**6.7**	<0.0001***
**Year: 2016**							
20 - <25	1	373	89.7	3	0.0	8.6	0.5058
25 - <50	164	6028	375.5	7.7	6.5	8.8	<0.001***
≥50	43	842	3829.6	3.8	2.6	4.9	<0.0001***
**Total**	**208**	**7286**	**1919.5**	**6.3**	**5.4**	**7.1**	<0.0001***

**Table 3:** Standardized mortality ratios by age strata, 2016–2018.

Age Group	Deaths among recently diagnosed	Total HIV diagnoses	Ukraine General Population Crude Mortality	Standardized Mortality Ratio	Lower 95% CI	Upper 95% CI	p-value
**Year: 2018**							
20–24	7	462	84.6	17.9	4.6	31.2	
25–29	21	1006	139.4	15.0	8.6	21.4	
30–34	86	1918	222.9	20.1	15.9	24.4	
35–39	125	2256	345.7	16.0	13.2	18.9	
40–44	120	1805	481.5	13.8	11.3	16.3	
45–49	85	1134	643.5	11.7	9.2	14.1	
50–54	73	719	903.9	11.2	8.7	13.81	
55–59	40	444	1274.0	7.1	4.9	9.3	
60–64	30	211	1883.3	7.6	4.9	10.3	
65–69	8	83	2742.9	3.5	1.1	6.0	
70–74	0	18	4116.2	--	--	--	
75–79	3	8	6595.6	5.7	0.00	12.1	
**Total**	**598**	**10064**	**1392.5**	**12.3**	**11.3**	**13.2**	<0.0001***
**Year: 2017**							
20–24	3	519	84.6	6.8	0.00	14.56	
25–29	12	1381	139.4	6.2	2.7	9.8	
30–34	36	2277	222.9	7.1	4.8	9.4	
35–39	55	2335	345.7	6.8	5.1	8.6	
40–44	58	1665	481.5	7.2	5.4	9.1	
45–49	40	1109	643.5	5.6	3.9	7.4	
50–54	31	650	903.9	5.3	3.4	7.1	
55–59	25	392	1274.0	5.1	3.0	7.0	
60–64	14	163	1883.3	4.6	2.2	7.0	
65–69	4	59	2742.9	2.5	0.1	4.9	
70–74	1	13	4116.2	1.9	0.00	5.5	
75–79	0	6	6595.6	--	--	--	
**Total**	**279**	**10569**	**1167.1**	**6.0**	**5.3**	**6.7**	<0.0001***
**Year: 2016**							
20–24	1	373	89.7	3	0.0	8.6	
25–29	10	920	142.2	7.6	2.9	12.4	
30–34	28	1530	225.5	8.1	5.1	11.1	
35–39	43	1651	360.8	7.2	5.1	9.4	
40–44	49	1188	489.9	8.4	6.1	10. 8	
45–49	34	739	658.9	7.0	4.6	9.3	
50–54	19	446	913.1	4. 7	2.6	6.8	
55–59	16	269	1296.6	4.6	2.3	6.8	
60–64	4	105	1981.8	1.9	0.1	3.8	
65–69	0	43	2822.1	--	--	--	
70–74	1	10	4285.1	2.3	0.0	6.9	
75–79	3	12	10671.3	2.3	0.0	5.0	
**Total**	**208**	**7286**	**1919.5**	**6.3**	**5.4**	**7.1**	<0.0001***

To assess representativeness, Chi-square goodness-of-fit compared the distribution percentages by both age and sex groups, between the UCPH and CPP databases (Tables 4 and 5).

**Table 4:** Comparison of UCPH and CPP data by year and age group.

Year	Data Source	Age Group				
		15–24	25–49	50+	Total	p-value
2015	UCPH	909 (7.1%)	10536 (81.7%)	1448 (11.2%)	12893	0.0071**
	CPP	288 (7.4%)	3133 (79.9%)	499 (12.7%)	3920	
2016	UCPH	884 (6.2%)	11604 (81.4%)	1761 (12.4%)	14249	0.648
	CPP	454 (6.2%)	6028 (81.8%)	885 (12.1%)	7367	
2017	UCPH	944 (6.1%)	12568 (80.7%)	2068 (13.3%)	15580	0.0003***
	CPP	622 (5.8%)	8767 (82.2%)	1283 (12.1%)	10672	
2018	UCPH	763 (4.9%)	12575 (80.2%)	2333 (14.9%)	15671	0.0773
	CPP	653 (5.1%)	10367 (80.7%)	1830 (14.2%)	12850	

**Table 5:** Comparison of UCPH and CPP data by year and gender.

Year	Data Source	Gender			p-value
		Men	Women	Total	
2015	UCPH	7466 (57.9%)	5427 (42.1%)	12893	0.0921
	CPP	2218 (56.6%)	1702 (43.4%)	3920	
2016	UCPH	8396 (58.9%)	5853 (41.1%)	14249	0.0001***
	CPP	4100 (55.7%)	3267 (44.3%)	7367	
2017	UCPH	9303 (59.7%)	6277 (40.3%)	15580	0.0902
	CPP	6287 (58.9%)	4385 (41.1%)	10672	
2018	UCPH	9559 (61%)	6112 (39%)	15671	0.5663
	CPP	7881 (61.3%)	4969 (38.7%)	12850	

There were, however, significant differences between two databases. Specifically, in 2015, the proportion of OPWH was higher in the CPP dataset (when it accounted for a lower proportion of all PWH in the national UCPH dataset), but in 2017, OPWH represented a lower proportion of individuals within the CPP dataset compared to UCPH. Last, in 2016 only, there was a higher proportion of women in the CPP dataset relative to the national UCPH dataset.

During the 2015–2018 period, heterosexual contact appears to be the predominant mode of transmission for all three age groups, but particularly for OPWH, where it accounts for 85% of new infections. Intravenous drug use (IVDU) is reported as the second most prevalent risk factor overall, but the proportion of new HIV infections due to IVDU among OPWH (14%) is about half that of younger adults (28%). Homosexual contact appears to account for 3% of all new HIV infections from 2015–2018, and for 1% of new infections among OPWH. (See S1 Table). Differences in proportions of new HIV infections reportedly acquired through (1) homosexual contact, (2) heterosexual contact, and (3) IVDU are significant (p<0.001) between age groups 15–24 years, 25–49 years, and ≥50 years.

## Discussion

To our knowledge, we report the first dedicated epidemiological assessment of trends among OPWH in Ukraine, a LMIC with both increasing HIV incidence and mortality [19]. We identified that older adults comprise an increasing proportion of newly identified PWH, clinically present with significantly more advanced HIV, and are at significantly increased risk of death within the first year of diagnosis. The ability to review two similar but distinct databases demonstrate that OPWH have been underrecognized in surveillance, treatment, and prevention efforts.

Concerningly, our analysis reveals the extraordinary proportion of adults ≥50 years in Ukraine who are diagnosed with advanced HIV. It suggests that these individuals have been chronically infected and have missed opportunities for testing and treatment. This finding echoes results from high-income European countries [31] where OPWH were more likely to present with advanced HIV infection (CD4<350 cell/mL). But, in Ukraine, a LMIC, the proportion of newly diagnosed OPWH with AIDS was markedly higher (43.8%) than in HICs of Western Europe (27.4%) [31]. This finding suggests a need for broadening beyond the targeted demographics and reappraising screening practices to ensure that OPWH are identified and engaged in treatment earlier in the course of disease. A realignment of the current HIV testing strategies is needed, one which involves routinely testing OPWH. Routine testing, including one-time testing of all people in the U.S., has narrowed the gaps in the HIV treatment cascade [44]. Current HIV testing strategies in Ukraine are based primarily on risk-based testing, where OPWH may either not perceive risk or under-report their risk, which has been documented in Ukraine [23, 45]. HIV testing campaigns have also focused on existing touchpoints, such as testing young women prior to giving birth, and younger adults of both genders when conscripting to military service, or being admitted to an educational institution. As a result, younger persons aged 15–49 years are much more likely to get tested for HIV. Only in the last decade has average life expectancy increased in Ukraine and there has consequently been increasing attention to addressing comorbidities of older adults. Such individuals may be in engaged in primary care, which should emerge as the new “touchpoint” for case-finding OPWH. Moving beyond HIV testing campaigns and risk-based testing will align with international goals to reduce stigma associated with HIV.

Thus, while tools are yet scarce to accurately assess risk for HIV among older adults, it may be helpful to triangulate it with other data sources, including with HCV or sexually transmitted infection data [23]. Further information is needed to characterize risk factors, current or not, rates of ART initiation and comorbidities to devise strategies to improve outcomes.

Consistent with studies from high-income settings [46, 47], we found significantly higher early mortality among newly diagnosed OPWH relative to age-matched counterparts ≥50 years in the general population who already have an expectedly higher age-related mortality. Mortality among all PWH was several-fold higher in 2018 than in the previous two years, possibly reflecting suboptimal scale-up of HIV treatment, though recent national numbers show increasing numbers of people on ART [17]. One potential explanation for this difference is that in Ukraine, clinicians anecdotally withhold ART from those with more advanced HIV disease afflicted with acute opportunistic infections, commonly comorbid tuberculosis, perhaps due to excessive concerns about immune reconstitution inflammatory syndrome (IRIS). Moreover, the higher risk for early death for OPWH relative to those 15–49 years, is particularly concerning as it undermines the 90-90-90 targets and undermines the 4^th^ 90 goal for achieving healthy and successful aging with HIV in LMIC like Ukraine. Next steps include understanding cause of death in this demographic group.

An important caveat must be made regarding information on HIV risk factors in Ukraine. While UCPH has started collecting self-reported data on HIV transmission risk factors since 2015, risk reporting may be not entirely accurate in Ukraine, especially in PWID and MSM [45, 48]. Furthermore, heterosexual contact transmission may subsume commercial sex work, that is not reported as a distinct risk factor. Our ongoing research is exploring this further (e.g., in-depth life histories and testing for HCV). Moreover, we have interviewed HIV physicians and out of reverence to older patients, they do not formally assess older patients’ alcohol and drug use, nor discuss sexually transmitted infections with them, which may reflect an element of ageism that had been reported in other global settings by OPWH [49], or age-based stereotype and discrimination (i.e., older people don’t engage in those kinds of behaviours). Future prospective studies need to examine transmission risk factors in OPWH.

The limitations of our analysis include the absence of CD4 and mortality information in the national statistical surveillance data. The ability to show that the clinical (CPP) database was, for the most part, similar to the national database is at least supportive of general trends observed. The findings are also limited by the use of aggregate rather than patient-level data. Programmatic, legal and economic restrictions limit access to patient-level data, but moving forward, using these findings to guide more creative use of these databases may spur innovations in clinical epidemiology for the country. Though we were able to demonstrate increased early mortality in OPWH relative to other age groups and to age-matched persons in the general population, we did not have access to causes of mortality, which would have been helpful to identify other causes of mortality (e.g., cardiovascular disease, cirrhosis, overdose), that may not have been directly linked to HIV. Ukraine’s commitment to the 90-90-90 targets will hopefully encourage more in-depth assessment of their data and use it for guiding HIV prevention and treatment. The findings here, for the first time, suggest the need to inform new strategies to address the 4^th^ 90 target (long-term survival with HIV and improved health-related quality of life) on the healthcare agenda. In line with the global HIV priorities listed in the WHO HIV 2016–2021 Strategy [2], increasing Ukraine’s capacity for collecting systematic data on OPWH and their comorbidities and mortality is imperative to timely development of effective and cost-effective testing and treatment interventions. The Ministry of Health of Ukraine has addressed this concern in their strategy for collecting PWH mortality data going forward, meaning better data in the future.

Notwithstanding these limitations, these data raise important concerns that require urgent attention. First, findings point to the need for Ukraine to scale up HIV testing and treatment efforts, especially in OPWH who may likely only be identified in routine primary care settings and perceived to be at minimal risk. The country has newly established a National Health System with a focus on primary care and routine testing is entirely aligned with this strategy where even addiction and HIV treatment are increasingly being transitioned to primary care clinics [50, 51]. While many high-income settings have moved away from risk-based testing and begun routine testing, this phenomenon has been vastly underappreciated in most LMICs where challenges in identifying HIV in key populations has evaded optimal HIV testing levels [34]. While these key populations still require focus, neglecting OPWH in LMICs will widen the gap in HIV care outcomes between high and low-income settings across the entire HIV care continuum, including health related quality of life [3].

As Ukraine struggles to balance both HIV detection and treatment deficits, including targeting individuals ≥50 years and their sexual partners, it will need to address the multiple co-morbidities associated with aging. The Ukrainian healthcare system is unprepared to provide complex care for the expanding number of OPWH with physical and mental aging health co-morbidities [52], and faces extra demands on its fragile resources from the ongoing Covid-19 epidemic [53]. Prior to the Covid-19 epidemic, most PWH received care in specialized treatment centres, which now have been reoriented to treat Covid-19 patients, with HIV care temporarily reduced to essential lab testing and ART dispensing. The transition from these specialty AIDS Centres represents a unique opportunity to strengthen primary care, including for HIV prevention and treatment. In the spirit of Building Back Better [54], there may be an opportunity for reorganizing HIV care post-Covid-19 lockdowns, to better integrate both primary and secondary care providers and develop patient-focused multi-disciplinary care strategies for OPWH, perhaps with inclusion of lay care workers [50, 55]. Such changes in healthcare delivery, however, should be guided by data on comorbidities, opportunistic infections, and causes of death among PWH.

Alongside scaling up its HIV testing strategies, especially in people over 50 years, in the context of Covid-19, Ukraine needs to plan for PWH over a lifetime to allow them to age with resilience and will require complex services. Going forward, this includes not only healthcare delivery models, including integrated care, but also elder care issues like allowing PWH to receive services in extended care facilities (which will emphasize the need to confront issues of HIV stigma, including among clinical staff) [56]. To reach the four 90 targets [57–59], it is essential that policy development for HIV care in Ukraine includes OPWH stakeholders, to develop truly patient-focused complex care.

### Conclusion

In Ukraine, OPWH account for an increasing proportion of new HIV diagnosis, more frequently have AIDS at diagnosis, and die earlier than their younger counterparts with HIV and earlier than age-matched counterparts in the general population. Innovative strategies to identify all PWH, including OPWH, are urgently needed and should be bonded to effective patient-linkage strategies should OPWH not link successfully. Once diagnosed and linked, it is crucial to plan for graceful aging over a lifetime for PWH, including those diagnosed later in life.

## Supporting information

S1 Table HIV risk transmission factors in Ukraine 2015–2018. (PDF)
