# Supplementary material for: An expanding HIV epidemic among older adults in Ukraine: Implications for patient-centered care
Source: PLoS One. 2021 Sep 30;16(9):e0256627. doi: 10.1371/journal.pone.0256627 (PMC8483339; doi:10.1371/journal.pone.0256627)
Supplement: S1 Table — (PDF) [file pone.0256627.s001.pdf]

Supplementary Table 5: HIV risk transmission factors in Ukraine 2015-2018.
Source: Ukrainian Center for Public Health supplementary forms Форма_1000а.

Transmission Category	Age Group 15-24 yo n (%)		25-49 yo n (%)		50+ yo n (%)		Total n (%)	
**Total (2015 - 2018)**								
	n=3500		***n=47283***		***n=7610***		***n=58393***	
**Homosexual contact**	427	12.20%	1314	2.78%	61	0.80%	1802	3.09%
**Heterosexual contact**	2716	77.60%	32368	68.46%	6464	84.94%	41548	71.15%
**IVDU**	336	9.60%	13554	28.67%	1057	13.89%	14947	25.60%
**Other**	16	0.46%	4	0.01%	5	0.07%	25	0.04%
**Unknown**	5	0.14%	43	0.09%	23	0.30%	71	0.12%
